# Single cell-type comparative metabolomics of epidermal bladder cells from the halophyte *Mesembryanthemum crystallinum*

**DOI:** 10.3389/fpls.2015.00435

**Published:** 2015-06-10

**Authors:** Bronwyn J. Barkla, Rosario Vera-Estrella

**Affiliations:** ^1^Southern Cross Plant Science, Southern Cross UniversityLismore, NSW, Australia; ^2^Departamento de Biologia Molecular de Plantas, Instituto de Biotecnología, Universidad Nacional Autónoma de MéxicoCuernavaca, Mexico

**Keywords:** salinity, salt-tolerance, metabolomics, halophyte, trichomes, epidermal bladder cells, Crassulacean acid metabolism, *Mesembryanthemum crystallinum*

## Abstract

One of the remarkable adaptive features of the halophyte *Mesembryanthemum crystallinum* are the specialized modified trichomes called epidermal bladder cells (EBC) which cover the leaves, stems, and peduncle of the plant. They are present from an early developmental stage but upon salt stress rapidly expand due to the accumulation of water and sodium. This particular plant feature makes it an attractive system for single cell type studies, with recent proteomics and transcriptomics studies of the EBC establishing that these cells are metabolically active and have roles other than sodium sequestration. To continue our investigation into the function of these unusual cells we carried out a comprehensive global analysis of the metabolites present in the EBC extract by gas chromatography Time-of-Flight mass spectrometry (GC-TOF) and identified 194 known and 722 total molecular features. Statistical analysis of the metabolic changes between control and salt-treated samples identified 352 significantly differing metabolites (268 after correction for FDR). Principal components analysis provided an unbiased evaluation of the data variance structure. Biochemical pathway enrichment analysis suggested significant perturbations in 13 biochemical pathways as defined in KEGG. More than 50% of the metabolites that show significant changes in the EBC, can be classified as compatible solutes and include sugars, sugar alcohols, protein and non-protein amino acids, and organic acids, highlighting the need to maintain osmotic homeostasis to balance the accumulation of Na^+^ and Cl^−^ ions. Overall, the comparison of metabolic changes in salt treated relative to control samples suggests large alterations in *M. crystallinum* epidermal bladder cells.

## Introduction

Single cell-type studies allow insight into the highly specific processes and functions of differentiated cells. In plants, studies into the role of specialized cells have been undertaken with a particular focus on those cells in which the isolation procedure is relatively uncomplicated. This includes free moving cell-types such as pollen grains, but also cells of the epidermis, such as root hairs, guard cells, and trichomes (Misra et al., 2014). Trichomes are widely conserved across the plant kingdom but are highly divergent in relation to morphology and function. They can be simple unicellular structures like the hairs of Arabidopsis, or complex multicellular appendages made up of differentiated basal, stalk, and apical cells; with the possibility of multiple trichome types co-existing on the same organ, as is observed for tomato (Kang et al., 2009). They produce, store and, in the case of glandular trichomes, secrete a diverse range of chemical compounds including polar and non-polar metabolites, which have been implicated in adaptive responses of the plant to their biotic and abiotic environments (Schilmiller et al., 2008). Trichomes can function to deter insects and herbivores, attract pollinators, aid in seed dispersal, regulate leaf temperature, but also to store unwanted xenobiotics including heavy metals and salts (Wagner et al., 1994).

In the Aizoaceae, specialized single cell trichomes called epidermal bladder cells (EBC) are a distinctive feature of this family (Klak et al., 2003). EBC of the succulent desert halophyte and facultative Crassulacean acid metabolism plant *Mesembryanthemum crystallinum* are abundant on leaves and stems from an early developmental age; however, cell morphology changes with development and metabolic/stress state of the plant (Adams et al., 1992). In young unstressed plants the EBC are small and tightly appressed to the leaf surface, whereas in adult salt-treated plants the EBC become enlarged and can be balloon- or sausage-like (Oh et al., 2015). They have been shown to accumulate high concentrations of sodium and thought to be important salt-adaptive features of the plant (Adams et al., 1998; Barkla et al., 2002). “Omics” approaches have helped to define a more encompassing role for the EBC, with proteomics and transcriptomics analysis suggesting these cells are metabolically active (Barkla et al., 2012), and show pronounced alternations in response to salt in a number of precisely defined pathways including significant changes in transcripts from networks representing Gene Ontology (GO) terms for ion transport, osmolyte accumulation, and stress signaling (Oh et al., 2015). Here we continue our systems wide integrative investigation of EBC in the facultative CAM plant *M. crystallinum* by carrying out non-targeted metabolite profiling of EBC extracts from plants under control and salinity treatment regimens to obtain a snapshot of EBC metabolism. Overall, the comparison of metabolic changes in salt treated relative to control samples suggested very large perturbations in metabolites between the treatment conditions and highlighted 13 significantly enriched biochemical pathways.

## Material and methods

### Plant materials and growth conditions

*M. crystallinum* L. plants were grown from seed in soil (MetroMix 510; Sun Gro Horticulture, Bellevue, WA) in a propagation tray as previously described (Barkla et al., 2009). Three weeks following germination, individual seedlings were transplanted to pots containing the soil mixture, with two plants per 15-cm-diameter pot. Plants were watered daily and one-half strength Hoagland's medium (Hoagland and Arnon, 1938) was supplied weekly. NaCl (200 mM) treatment was initiated 6 weeks after germination for a period of 14 d. Plants were grown in a glasshouse under natural irradiation and photoperiod, with the greenhouse photosynthetic photon flux density reaching a peak value of 1300 mmol m^−2^ s^−1^ during the middle of the day. Temperature was maintained at 25°C ± 3°C.

### Extraction of bladder cell extract

Bladder cell extract was obtained from individual cells on the leaf abaxial epidermal surface and stems by vacuum aspiration of the cell contents using a fine gage insulin needle (27 G, 13 mm) attached to a collection reservoir maintained on ice. The needle was oriented horizontally to the leaf or stem axis to avoid removing sap from underlying tissue and the procedure was visualized using a Nikon SMZ645 stereo microscope equipped with a dual arm Nikon MKII fiber optic light source (Nikon, México). The extracted liquid from approximately 2000 EBC estimated by counts of cell yield per leaf/stem section from a single control or salt-treated plant was pooled to obtain a single biological replicate. Seven biological replicates were collected per treatment. Plants were maintained in the dark prior to extraction and collection was undertaken early in the morning.

### Sample preparation for metabolomics analysis

Bladder cell extract was aliquoted into 1.5 ml Eppendorf tubes and the extract was evaporated in a Labconco Centrivap concentrator (Kansas City, MO, USA) to complete dryness. The dried extract was then resuspended in the indicated pre-chilled (to –20°C) nitrogen degassed extraction solution. Samples were vortexed for 10 s, and placed on an orbital shaker at 4°C for 6 min and finally dried in the Labconco Centrivap cold trap concentrator to complete dryness prior to derivitization. Two experimental replicates were obtained for each biological replicate.

### Gas chromatography time-of-flight mass spectrometry (GC-TOF)

GC-TOF was performed by West Coast Metabolomics at the UC Davis Genome Centre (Davis CA, USA). Mass spectrometry was performed using a Pegasus III Time Of Flight (TOF) mass spectrometer (LECO, St. Joseph, MI, USA) coupled to an Agilent 6890N gas chromatograph (Agilent Technologies) equipped with a Gerstel autosampler, including a MPS PrepStation and Automated Liner EXchange (ALEX) (Gerstel, Muehlheim, Germany). Liner was changed after every 10 samples, (using the Maestro1 Gerstel software vs. 1.1.4.18). Before and after each injection, the 10 μl injection syringe was washed three times with 10 μl ethyl acetate. The Gas Chromatograph was equipped with a 30 m long, 0.25 mm i.d. Rtx-5Sil MS column (0.25 μm 95% dimethyl 5% diphenyl polysiloxane film) with additional 10 m integrated guard column (Restek, Bellefonte PA, USA). Pure helium (99.9999%) with built-in purifier (Airgas, Radnor PA, USA) was set at constant flow of 1 ml/min. The oven temperature was held constant at 50°C for 1 min and then ramped at 20°C/min to 330°C at which it was held constant for 5 min. The injector was set to a temperature of 250°C and was used in split mode with 10:1 ratio. The injection volume was 1 μl. The transfer line temperature was set to 280°C (GC re-entrant temperature). The mass spectrometer, controlled by Leco ChromaTOF software vs. 2.32 (St. Joseph, MI, USA), was operated in positive EI mode. All EI spectra were collected using an electron energy of 70 eV, trap current of 250 mA, emission current of 600 mA, filament current of 4.5 A, and source temperature of 250°C. Acquisition rate was 17 spectra/s, with a scan mass range of 85–500 Da. Spectra were deconvoluted from co-eluting peaks with ChromaTOF, this software detects peaks in an unbiased way and exports one deconvoluted spectrum per peak.

### Metabolite identifications

Following acquisition of data, a BinBase relational database system was used to allow for automated metabolite annotation. Metabolites were grouped into either a list of, “identified compounds” or a list of “unknown compounds” and assigned as previously described Lee and Fiehn (2008). Accordingly both groups were unambiguously assigned using the identification criteria of retention index and mass spectrum to BinBase identifier numbers. Additional confidence criteria were given by mass spectral metadata, using the combination of unique ions, apex ions, peak purity, and signal/noise ratios. Each BinBase identifier was routinely matched against the Fiehn lab mass spectral library (Fiehn et al., 2005). For named BinBase compounds, PubChem numbers and KEGG identifiers were added. In addition, for all reported compounds (identified and unknown metabolites) the quantification ion and the full mass spectrum encoded as string is reported.

### Data analysis and accessibility

Statistical analyses were conducted on natural logarithm transformed metabolic parameters, and data summaries are presented for raw data values. HCA of samples, and PCA were implemented on autoscaled data (mean centered and scaled by the standard deviation, z-scaled). The data obtained in this study will be accessible at the NIH Common Fund's Data Repository and Coordinating Center (supported by NIH grant, U01-DK097430) website, http://www.metabolomicsworkbench.org.

## Results

### Extraction of metabolites

Untargeted, global metabolite profiling studies aim to analyse as many small-molecular-weight species with a single sample extraction protocol as possible. However, due to the large variety of metabolites with different chemical stability, solubility, and polarity, the choice of sample-preparation method can greatly influence the observed metabolite content of a cellular extract. Ideally the method should be as non-selective as possible to ensure maximal depth of coverage. We first established the optimal sample extraction protocol for GC-TOF of EBC extracts that would allow us to obtain the greatest coverage of the EBC metabolome. Three solvent extraction methods were tested: 9:1 methanol/chloroform, 5:2:2 methanol/chloroform/water and 10:3:1 methanol/chloroform/water. Varying the ratio of aqueous and organic solvents can influence the recovery of polar and non-polar metabolites. Supplementary Table 1 shows the metabolite profile of EBC's extracted using the different solvent regimes and two different volumes (200 and 500 μl). In all extracts we were able to identify 668 different molecular features (175 known and 493 unknown). However, the amounts extracted varied between the different procedures (Supplementary Table 1) suggesting a variation in recovery efficiency similar to what was shown for yeast metabolite extraction (Tambellini et al., 2013). Based on this information we selected 5:2:2 methanol/chloroform/water solvent to water ratio which has been shown to extract comparatively high levels of both polar metabolites and non-polar fatty acids (Lee and Fiehn, 2008).

### Alteration in metabolites in salt-treated EBC samples

**Figure 1:**
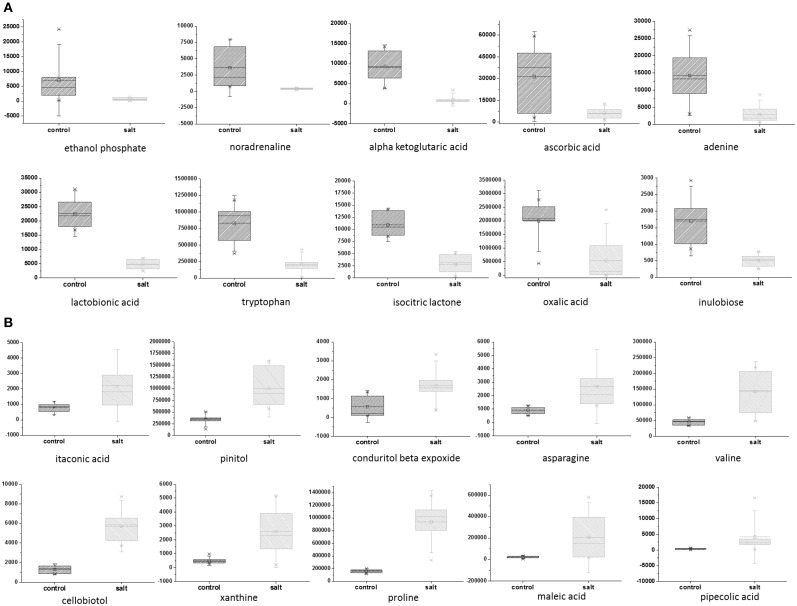
**Top 10 down- and up-regulated metabolites in EBC extracts from salt-treated plants compared to control plants**. Metabolites which were significantly down-regulated (**A**) and upregulated (**B**), as determined by a two-sample *t*-Test. The probability or significance level for the test statics (i.e., *p*-values) were adjusted for the multiple hypotheses tested to allow for a maximum 5% probability (*q* = 0.05) of false positives. Box and whiskers plots showing natural logarithm transformed values are used to visualize the group means and standard deviations.

To explore the effect of salinity on EBC metabolite levels, samples from 7 independent biological replicates for untreated and salinity–treated plants were collected, extracted with 5:2:2 methanol/chloroform/water, and metabolic parameters (194 known and 722 total molecular features) were compared Supplementary Table 2. A two-sample Student's *t*-Test was used to assess the significance of the difference between the control and salt-treated samples. The probability level for the *p*-values were adjusted to allow for a maximum 5% probability (*q* = 0.05) of false positives (Benjamini and Hochberg, 1995). The FDR was also directly estimated as the *q*-value, for all comparisons (Klaus and Strimmer, 2012). Statistical test results are reported along with metabolite averages, standard deviations and fold changes of means in Supplementary Table 2. A total of 352 significantly differing metabolites (268 after correction for FDR) were identified (Supplementary Table 2). Of the known metabolites, 28 showed a statistically significant down-regulation of more than 1.5 fold (Table 1) and 28 metabolites showed a statistically significant up-regulation of more than 1.5 fold (Table 1). The top 10 known, significant down- and up-regulated metabolites are depicted in box plots (Figures 1A,B). Of the significantly changing unknown metabolites, nine showed more than a 5-fold increase in the salt-treated samples compared to the control samples, while an additional 13 were shown to be significantly down-regulated by more than 5-fold (Supplementary Table 2).

**Table 1:** **Metabolites which were significantly down- or up-regulated by more than 1.5-fold in EBC extracts from salt-treated plants compared to control plants**.

**Metabolite name**	**Metabolite classification**	**Fold change^a^**	***p*-values^b^**	**Adjusted *p*-values^c^**	***q*-values^d^**
**DOWN-REGULATED**					
Tyramine	amine	0.36	0.00008	0.00151	0.00035
Putrescine	amine	0.40	0.00128	0.00947	0.00221
Phenylethylamine	amine	0.44	0.01377	0.04125	0.00961
Ornithine	amine	0.44	0.01629	0.04700	0.01095
Tryptophan	amino acid	0.25	0.00739	0.02681	0.00624
Histidine	amino acid	0.49	0.00559	0.02307	0.00537
Noradrenaline	hormone	0.11	0.00165	0.01048	0.00244
Cysteine-glycine	non-protein amino acid	0.32	0.00150	0.00982	0.00229
Kynurenine	non-protein amino acid	0.33	0.00000	0.00024	0.00006
Alpha ketoglutaric acid	organic acid	0.12	0.00002	0.00047	0.00011
Ascorbic acid	organic acid	0.20	0.01459	0.04317	0.01005
Isocitric lactone	organic acid	0.25	0.00229	0.01342	0.00312
Oxalic acid	organic acid	0.27	0.01407	0.04197	0.00977
2-hydroxyglutaric acid	organic acid	0.39	0.00065	0.00582	0.00136
Dihydroxymalonic acid	organic acid	0.44	0.00073	0.00618	0.00144
2-hydroxyadipic acid	organic acid	0.46	0.00299	0.01574	0.00367
Ethanol phosphate	other	0.09	0.00568	0.02319	0.00540
Glycerol-alpha-phosphate	phosphate	0.38	0.00007	0.00129	0.00030
Adenine	purine	0.20	0.00115	0.00880	0.00205
Uridine	pyrimidine	0.31	0.00664	0.02539	0.00591
Inulobiose	sugar	0.30	0.00011	0.00164	0.00038
Maltose	sugar	0.37	0.00064	0.00582	0.00136
Cellobiose	sugar	0.42	0.00072	0.00618	0.00144
Beta-gentiobiose	sugar	0.50	0.00026	0.00289	0.00067
Lactobionic acid	sugar acid	0.21	0.00000	0.00006	0.00001
Galactonic acid	sugar acid	0.49	0.00028	0.00303	0.00071
Erythritol	sugar alcohol	0.30	0.00030	0.00311	0.00072
Ribitol	sugar alcohol	0.47	0.00165	0.01048	0.00244
**UP-REGULATED**					
Triethanolamine	Amine	2.15	0.00239	0.01370	0.00319
Threonine	Amino acid	2.20	0.00011	0.00163	0.00038
Methionine	Amino acid	2.49	0.00129	0.00947	0.00221
Adenosine	Amino acid	2.59	0.01693	0.04775	0.01112
Asparagine	Amino acid	2.95	0.00219	0.01320	0.00307
Valine	Amino acid	3.09	0.00189	0.01166	0.00272
Sinapyl alcohol	Cell wall lignification	1.79	0.00674	0.02539	0.00591
1-monopalmitin	Fatty acid	1.78	0.02282	0.05604	0.01305
Dodecanol	Fatty alcohol	2.22	0.00335	0.01670	0.00389
4-hydroxybutyric acid	Non-protein amino acid	1.62	0.01725	0.04807	0.01120
Beta-alanine	Non-protein amino acid	2.27	0.00778	0.02766	0.00644
N-methylalanine	Non-protein amino acid	2.50	0.00258	0.01423	0.00331
Proline	Non-protein amino acid	5.94	0.00002	0.00047	0.00011
Pipecolic acid	Non-protein amino acid	12.71	0.00429	0.01923	0.00448
Dehydroascorbic acid	Organic acid	2.36	0.00267	0.01462	0.00340
Itaconic acid	Organic acid	2.81	0.00777	0.02766	0.00644
Maleic acid	Organic acid	9.31	0.02157	0.05427	0.01264
Butyrolactam	Other	2.36	0.01847	0.04975	0.01159
1-methyl-1-(4-methyl-3-cyclohexen-1-yl)ethoxy	Other	2.69	0.00410	0.01892	0.00441
Xanthine	Purine	5.28	0.01299	0.03990	0.00929
Uracil	Pyrimidine	2.53	0.01100	0.03497	0.00815
Xylose	Sugar	1.73	0.00893	0.03042	0.00709
Fructose	Sugar	2.01	0.01239	0.03873	0.00902
Glycerol	Sugar alcohol	1.82	0.00094	0.00743	0.00173
1,2-anhydro-myo-inositol	Sugar alcohol	2.68	0.00005	0.00095	0.00022
Pinitol NIST	Sugar alcohol	2.91	0.00033	0.00338	0.00079
Conduritol beta expoxide	Sugar alcohol	2.92	0.01335	0.04081	0.00950
Cellobiotol	Sugar alcohol	4.32	0.00000	0.00007	0.00002

a *Fold change—mean salt-treated value/mean control value*.

b *p-values—Significance levels for the two-sample Student's t-Test were computed for assessing the significance of difference between salt-treated samples and control samples*.

c *Adjusted p-values—the significance level for the test statistics were adjusted for the multiple hypotheses tested to allow for a maximum 5% probability of false positives (pFDR-adjusted p-values)*.

d *q-values—estimate of the FDR for all comparisons*.

### Hierarchical cluster analysis

**Figure 2:**
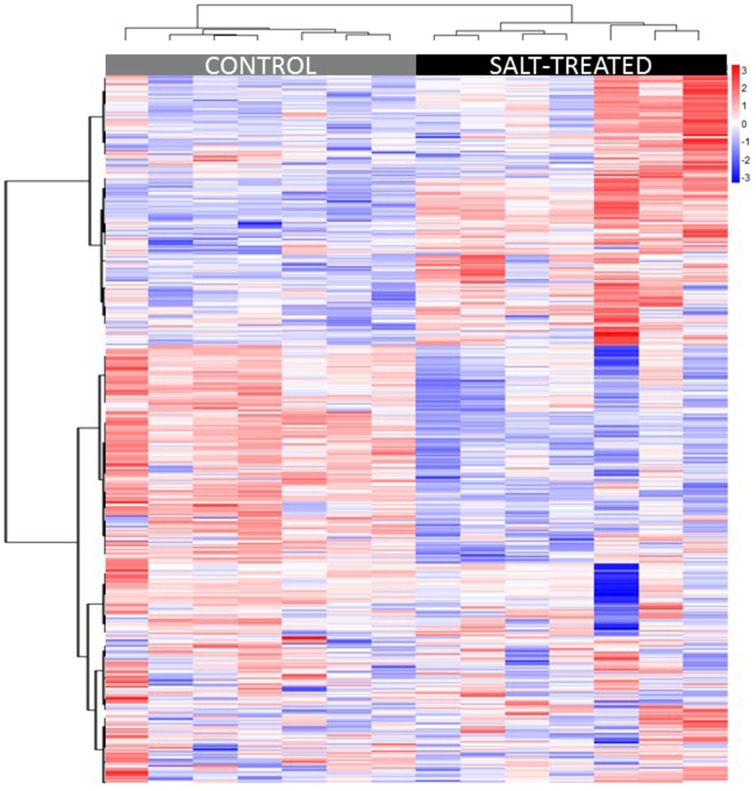
**Heatmap displaying similarities between biological samples organized by HCA**. Multivariate sample similarities, displayed as a heatmap in which columns represent samples and rows metabolites. The heatmap visualization is used to encode individual measurements for each sample (autoscaled) as colors (red, relative increase; blue, relative decrease), as indicated by the color bar at top right of figure, all of which are organized using HCA. Metabolites are ordered on the heatmap in the order they appear in the list in Supplementary Table 2.

Hierarchical cluster analysis (HCA) was used to group samples and metabolites based on similarities in auto scaled values and correlations, respectively (Sugimoto et al., 2012). Distance was calculated based on the Euclidean method, linkage was done using the Ward method (Ward, 1963), and variable similarities were based on Spearman correlations. Multivariate sample similarities, displayed as a heatmap, are shown in Figure 2. Separation of experimental treatments or sample classes into non-overlapping clusters suggests large metabolic differences between the comparisons. While separation of similar classes of samples into clusters may suggest high biological or analytical variability.

### Principal component analysis

**Figure 3:**
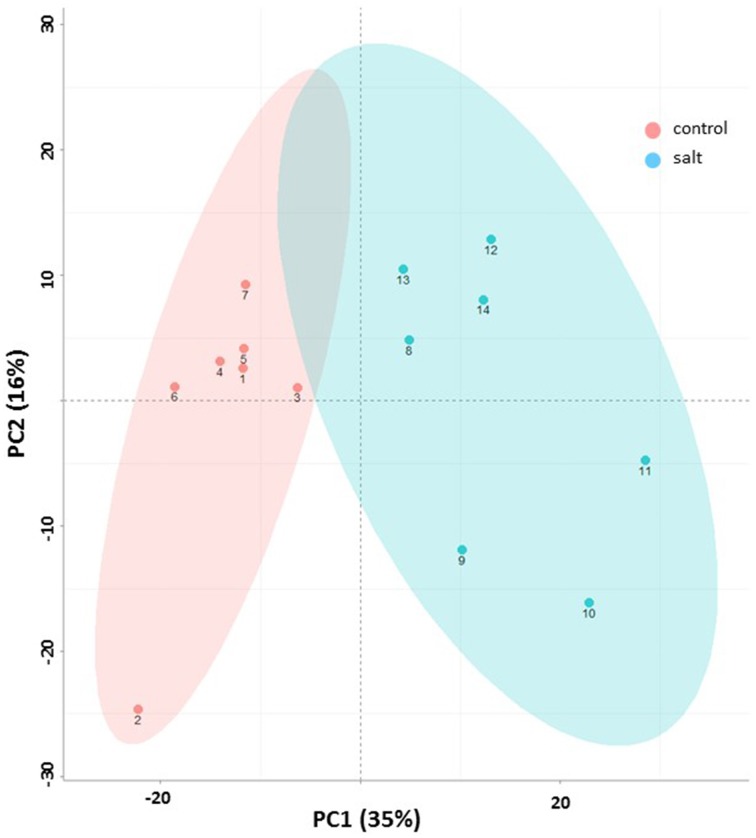
**Metabolic changes associated with salinity**. Principle component analysis (PCA) plot (x – first component, y – second component) of control (pink) and salt-treated (blue) EBC extract of GC/MS based metabolites. Symbol numbers represent the different biological replicates (1–7, control replicates; 8–14, salt-treated replicates). Colored ellipses (based on the Hotelling's T2 95% confidence interval) are used to display experimental group scores.

Principal component analysis (PCA) provided an unbiased evaluation of the data variance structure. Figure 3 shows the PCA for the dataset of EBC extracts from control and salt treated plants for the first two principal components. As can be observed the experiments are clearly separated into two distinct groups which reflect the treatment conditions. The first principal component (PC1)—explained the greatest variance (35%) across the data and separates the samples based on treatment. The second principal component (PC2) separated the components based on sample replicates and accounted for 16% of the variance.

### Biochemical pathway enrichment

Pathway enrichment analysis was used to identify if the significantly changed metabolites, between control and salt-treated EBC samples, were significantly enriched in members of biochemical pathways as defined by the KEGG Database. Significant enrichment in KEGG pathways was identified using MBRole (Chagoyen and Pazos, 2011). The hypergeometric test coupled with adjustment for FDR (Benjamini and Hochberg, 1995) was used to identify 13 significantly enriched pathways (Table 2); which included aminoacyl-tRNA biosynthesis, glutathione metabolism, biosynthesis of alkaloids derived from histidine and purine, C_5_-branched dibasic acid metabolism, biosynthesis of alkaloids derived from ornithine, lysine and nicotinic acid, beta-alanine metabolism, biosynthesis of plant hormones, biosynthesis of phenylpropanoids, butanoate metabolism, galactose metabolism, cyanoamino acid metabolism and glyoxylate and dicarboxylate metabolism.

**Table 2:** **Pathways which are significantly enriched for the metabolites**.

**Metabolic pathways**
Aminoacyl-tRNA biosynthesis
Glutathione metabolism
Biosynthesis of alkaloids derived from histidine and purine
C5-Branched dibasic acid metabolism
Biosynthesis of alkaloids derived from ornithine, lysine and nicotinic acid
beta-Alanine metabolism
Biosynthesis of plant hormones
Biosynthesis of phenylpropanoids
Butanoate metabolism
Galactose metabolism
Cyanoamino acid metabolism
Glyoxylate and dicarboxylate metabolism

*Significant using adjusted p-value <= 0.05. Arabidopsis thaliana was used as a background set*.

## Discussion

Within the plant kingdom it is believed there are an estimated 200,000 metabolites (Fiehn, 2002), however species specificity, specialized organ, tissue and cellular distribution, spatiotemporal factors, and rapid metabolite turnover, combined with the difficulty to capture and detect the metabolites, severely limits the number of metabolites that can be detected in an organism at any one time. Employing multiple analytical approaches can increase the number of metabolites detected, but also simply optimizing the extraction protocol can influence the success of detection. In this study, following extraction optimization steps, we were able to identify a total of 722 metabolites in single cell-type EBC extracts consisting of 194 known and 528 unidentified molecular features. The amount of known metabolites detected in EBC extracts was significantly higher than previous reports of single cell-type global metabolite profiling of leaf trichomes, including glandular trichomes from *Solanum* (119 known compounds/LC-MS/3:3:2 propanol:acetonitrile:water) and simple trichomes from Arabidopsis (119 compounds/GC-TOF/ 1:2.5:1 chloroform:methanol:water) (Ebert et al., 2010; McDowell et al., 2011). This may be a result of selecting a more optimized extraction protocol, or related to cell-type specific metabolic diversity, but could also be due to improvements in bioinformatics pipelines for the chemical assignment of unknown ions in metabolome data.

**Figure 4:**
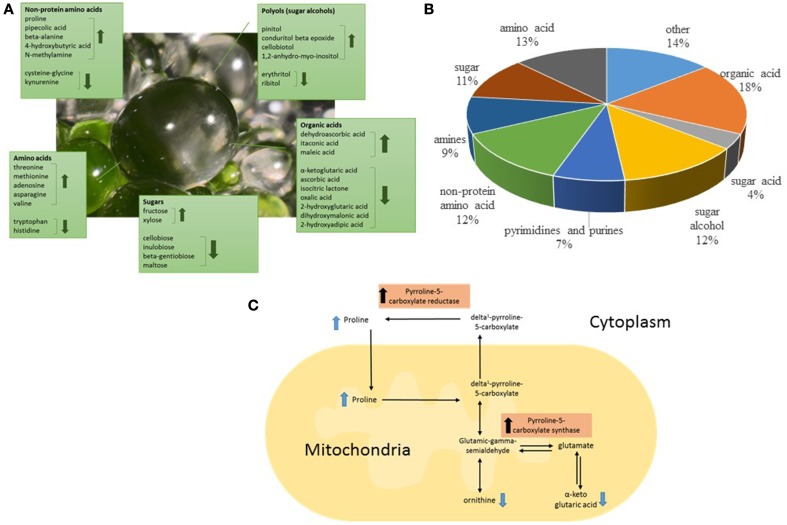
**Classification of the significantly salt-regulated EBC metabolites based on their known function and chemical attributes**. **(A)** A large proportion of the identified metabolites could have roles as compatible solutes in the EBC, including those classified as amino acids, sugars, organic acids, and polyols. **(B)** The pie graph shows the relative composition of the identified metabolites. **(C)** Representation of proline biosynthesis pathway showing the location in the pathway of identified metabolites and indicating the enzymes in the pathway which have been shown to be up-regulated in RNAseq analysis of EBC (Oh et al., 2015).

In order to gain greater insight into the biochemical function and underlying biological roles of the EBC we studied the effect of salt treatment on the EBC metabolites. Statistical analysis detected significant changes in 37% of the total metabolites (Supplementary Table 2). Of the 194 known metabolites identified we were able to detect significant changes in 57 (30%) of these upon salt-treatment (Table 1), suggesting an extensive alteration in the metabolome in response to salinity. The most highly increased metabolite (more than 12 fold) was the non-protein amino acid pipecolic acid, shown to be a critical regulator of inducible plant immunity and resistance to bacterial pathogens (Návarová et al., 2012), but also known to accumulate upon salt stress in *Limonium vulgare* (Stewart and Larher, 1980). Pipecolic acid is an analog of proline, sharing a similar chemical structure (Ganapathy et al., 1983). Proline is another non-protein amino acid that showed high accumulation in salt stressed EBC (Table 1). In fact, more than 50% of the metabolites that show significant changes in the EBC, can be classified as compatible solutes and include sugars, sugar alcohols, protein and non-protein amino acids, and organic acids (Figures 4A,B, Table 1) (Slama et al., 2014). This data correlates well with gene ontology enrichment analysis of differentially expressed transcripts in the EBC, with GO term networks “small molecule metabolic process” and “carboxylic acid metabolic process”, significantly enriched in the salt-treated EBC transcripts as compared to the control (Oh et al., 2015). The accumulation of osmotically active non-toxic organic compounds in the cytosol increases the cellular osmotic potential to provide a balance between the cytoplasm and the vacuolar lumen, which, in *M. crystallinum* can accumulate up to 1 M Na^+^ (and Cl^−^) (Adams et al., 1992). Both proline and pinnitol have been shown to increase previously in EBC from salt-treated plants (Paul and Cockburn, 1989; Adams et al., 1998), and RNAseq results suggested that salt treatment induces metabolic pathways that lead to synthesis, accumulation, transport and conversion of compatible solutes (Oh et al., 2015). Specifically, increases in pinitol can be linked to increases in transcripts encoding myo-inositol-1-phosphate synthase and myo-inositol O-methyltransferase 1, two key enzymes in the pathway leading to pinitol synthesis via ononitol, which were highly abundant and significantly upregulated in the EBC transcriptome (up to 170-fold) following salinity treatment (Oh et al., 2015). Expression of transcripts encoding two key proline biosynthesis enzymes, delta-1-pyrroline-5-carboxylate synthase and pyrroline-5-carboxylate reductase were also salt-induced in the EBC (Oh et al., 2015) and link changes in other metabolites, including ornithine and alpha ketoglutaric acid (Table 1) to proline accumulation (Figure 4C).

A high number of organic acids were identified as significantly altered in the EBC following salt-treatment (Table 1, Figure 4B). As well as their possible function as metabolically active solutes for osmotic adjustment, organic acid metabolism is of fundamental importance at the cellular level for several biochemical pathways, including as photosynthetic intermediates in CAM (Cushman and Bohnert, 1997), and for the formation of precursors for amino-acid biosynthesis (López-Bucio et al., 2000). It is likely that changes in abundance of organic acids reflects all these roles. One of the most highly altered organic acids was the C_4_ dicarboxylic acid maleate (maleic acid, 9.41-fold increase), a trans-isomer of fumaric acid, it is considered an uncommon plant metabolite (Fiehn et al., 2000), and its biological role is not clear, other than as a possible citric acid cycle intermediate. It was shown to be negatively correlated with Na^+^ concentration in roots from salt-treated barley seedlings (Wu et al., 2013), and was a cold shock inducible metabolite identified in Arabidopsis shoots (Kaplan et al., 2004), as well as being identified in Arabidopsis and cotton seed trichomes (Ebert et al., 2010; Naoumkina et al., 2013); but these reports give little insight into its function in the plant.

Amino acids are the predominant form of solute accumulated by a phylogenetically diverse range of salt-tolerant organisms such as salt-tolerant bacteria, halophytes, marine invertebrates, and hagfishes (Yancey et al., 1982). However, only selected protein amino acids appear to be utilized. In EBC there was an overall net increase in amino acids (Table 1), with the highest increase (approx. 3-fold) observed for asparagine and valine. Both these amino acids have been reported to increase upon salt-treatment (Rabe, 1990; Martinelli et al., 2007; Nedjimi, 2011; Zhang et al., 2011) and are implicated as nitrogen stores during stress to maintain metabolic homeostasis (Mansour, 2000; Rabe, 1990).

Both ascorbic acid and its oxidized form dehydroascorbic (DHA) acid are significantly altered in salt-treated EBC, with ascorbate decreasing and dehydroascorbic increasing. In plants ascorbate is the most abundant water-soluble antioxidant (Smirnoff, 2000) and participates in cellular redox state homeostasis by maintaining ROS below toxic levels but also through temporal–spatial coordination of ROS for a multitude of signaling pathways (Baxter et al., 2013). Salinity stress results in the increased production of ROS (Abogadallah, 2010) and the recycling of ascorbic acid may be critical for maintaining ROS at a level within the EBC that minimizes oxidative damage but permits signaling function (Gallie, 2013). Ascorbic acid reacts with ROS and is oxidized to the radical MDHA which can rapidly disproportionate non-enzymatically to produce dehydroascorbic acid and recycled ascorbic acid (Smirnoff, 2000). The accumulation of dehydroascorbic acid in the EBC may reflect this recycling process. Oxidation of ascorbate has also been implicated in cell growth through the generation of MDHA radicals, and downstream stimulation of the plasma membrane proton ATPase which acidifies the extra cellular space resulting in cell wall loosening (Kato and Esaka, 2008).

Sinapyl alcohol, one of the main building blocks of lignin (Vanholme et al., 2010) is increased in the EBC from salt-treated plants. Lignin is deposited in the secondary cell walls of all vascular plants forming cross-links with other cell wall components to create rigidity. Increased lignification may be essential for the structural integrity of the swelling bladder cells to provide increased support as their volume increases with the accumulation of ions and water. Additionally, increases in the fatty alcohol dodeconol, a component of the waxy cuticular film that is present on the aerial surface of plants (Kunst and Samuels, 2009) would help to protect the swelling bladder cells, limiting non-stomatal water loss, while increases in fatty acids, such as is observed for monopalmitin under salt-stress, would serve as precursors to fatty alcohol synthesis in the EBC (Kunst et al., 2006). Salt induced increases in transcripts for enzymes involved in biosynthesis of plant cuticular wax, including fatty acid hydrolase, were also observed in EBC transcriptome (Oh et al., 2015).

Comparison of global metabolic changes upon salt stress with other halophytes, including the legume *Lotus creticus* (Sanchez et al., 2011), and the salt-tolerant Arabidopsis relative *Thellungiella salsuginea* (Lugan et al., 2010), highlights a similarity in the classes of metabolites which show highest alteration upon salinity treatment, in particular, sugars, sugar alcohols, organic acids, and amino acids. This is not surprising as compatible solute synthesis is a well-documented tolerance response in most plants to salinity (Hasegawa et al., 2000), but there was little agreement in the specific metabolites that change, implying the existence of differential metabolic arrangements between species and possibly cell types, to compensate for ion imbalance (Flowers and Colmer, 2008).

Of the chemically unidentified metabolites, 21 showed changes of more than 5-fold in the salt-treated EBC samples compared to the control samples. These could potentially make important contributions to the cell-type specific adaptations to salt in *M. crystallinum* EBC. Recent advances in linking functionally identified genes to unknown metabolites by genome wide association studies, integrating the data from genetic associations and metabolic networks with biochemical pathway information, has been successful in both humans (Krumsiek et al., 2012) and plants (Luo, 2015) for annotation of unidentified metabolites and will provide a means in the future to accelerate research in this area.

### Conflict of interest statement

The authors declare that the research was conducted in the absence of any commercial or financial relationships that could be construed as a potential conflict of interest.
