# Supplementary figures and images for: Single cell-type comparative metabolomics of epidermal bladder cells from the halophyte Mesembryanthemum crystallinum
Source: Front Plant Sci. 2015 Jun 10;6:435. doi: 10.3389/fpls.2015.00435 (PMC4462104; doi:10.3389/fpls.2015.00435)

## Slide 1
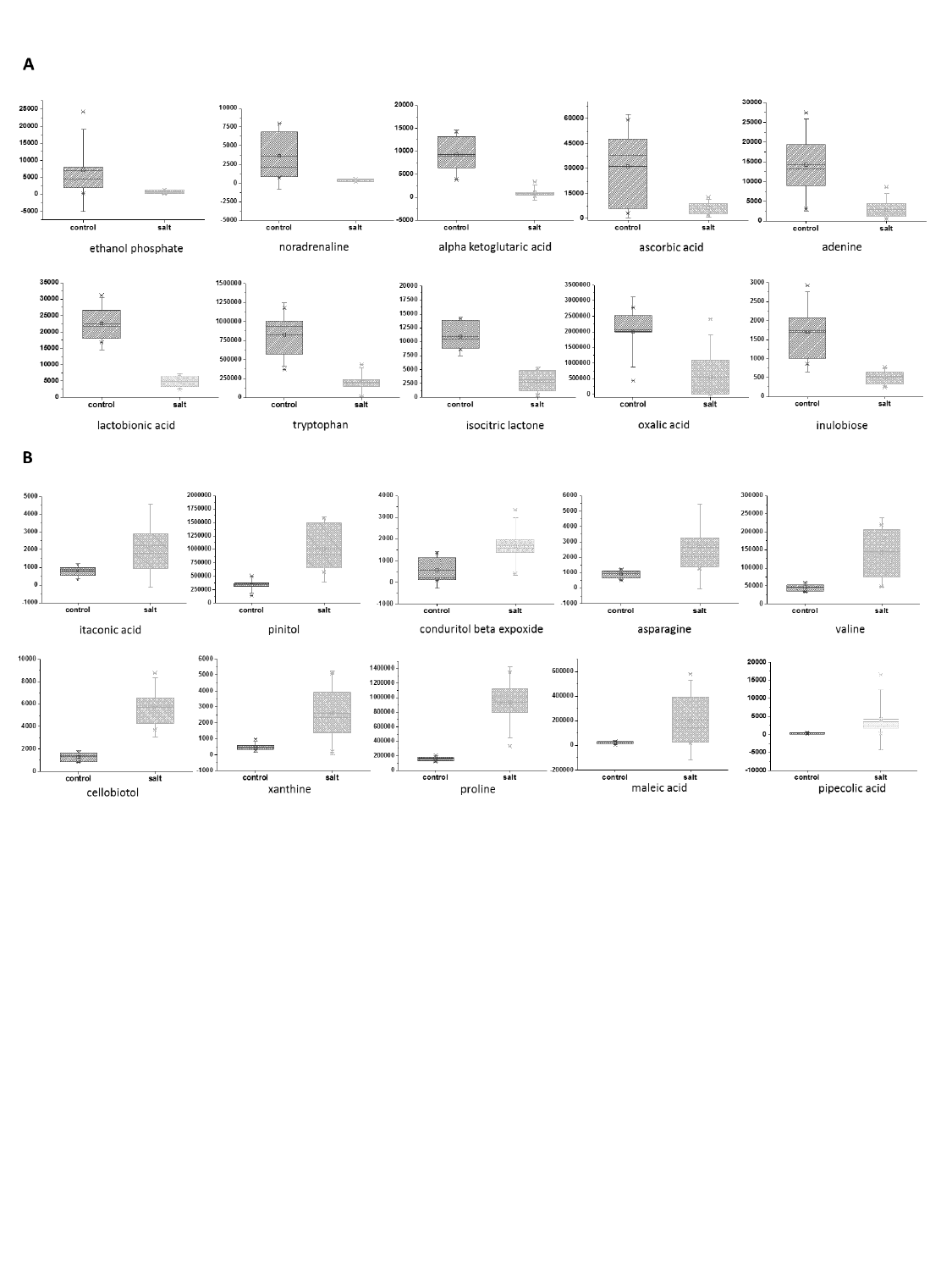

Supplement: Supplementary file 3 [file Presentation_1.PPTX]
